# Are gastrointestinal signals the principal guides to human appetite and energy balance?

**DOI:** 10.18103/mra.v11i1.3548

**Published:** 2023-01-31

**Authors:** Katarina T. Borer

**Affiliations:** School of Kinesiology, The University of Michigan, Ann Arbor, Ml 48109, USA

## Abstract

In view of the exponential rise of global obesity in the past three quarters of the century, it is useful to examine what is driving this change and what approaches can curb it. The chief drivers of weight gain are, on one hand our misunderstanding of the mechanisms controlling energy balance, and, on the other, reliance on current, potentially misleading conflicting scientific opinions and government policies regarding the controls of human appetite. This review outlines the evidence that: (1) there is no direct bioenergetic feedback from energy metabolism or energy stores to the brain mechanisms guiding feeding and energy expenditure, (2) human appetite is controlled by signals originating from an empty or full stomach, food palatability and opportunities to eat as well by the rate of food absorption, that (3) humans bear a genetic burden of having high ability and capacity to store fat and mechanisms that curb body- mass and fat loss, (4) humans are motivated to overconsume while maintaining low energy expenditure, and (5) commercial interests of food businesses marketing highly palatable foods, and wide-spread mechanization of living tasks and urban design reduce the need for physical work and movement. The non-pharmacological and non-surgical solutions to obesity involve an understanding of human genetic impediments and environmental obstacles to maintaining healthy weight, coupled with deliberate corrective or preventive behaviors, such as understanding and using gastrointestinal tract signals that provide sufficient, albeit subtle, cues for sensible food intake, and using daily weight monitoring and activity tracking devices to record and motivate healthy levels of physical activity.

## INTRODUCTION

The exponential rise of excess weight and obesity in the past three quarters of a century poses a global health problem^1^. Prevalence of obesity in 200 countries world-wide tripled between 1975 and 2014 and continues to rise^2,3^. In the USA, 65% adults were overweight, and 42% were obese in 2018^4^. Being overweight or obese risks developing a cluster of cardiovascular (CVD) and metabolic diseases^5,6^. Because of the difficulty of reducing excess weight long-term by lifestyle approaches^7,8^, obesity has been characterized as a chronic, relapsable disease by American Medical Association in 2013 and by European Health Commission in 2021^9^.

This review provides evidence that individuals have the agency to prevent and reduce obesity by paying attention to the gastrointestinal (GI) signals as the principal guides to human appetite and energy balance and by being cognizant of human physiological limitations and environmental obstacles to maintaining healthy weight. The endogenous mechanisms for elicitation of hunger and satiation are outlined first. Human physiological limitations, such as large inborn capacity to store fat and enlarge stomach size, social and environmental factors leading to overeating, reasons for inadequate motivation to expend energy, and multiple defenses against body fat loss, are discussed next. Finally, suggestions are provided for successful use of hunger and fullness signals and for avoidance of environmental interference, while deliberately controlling meal taking and enjoying exercise energy expenditure.

## EVIDENCE-BASED THESIS

### 1. Endogenous elicitation of hunger.

Our sensation of hunger is an inborn signal and key motivator to seek food and initiate eating. While eating is accompanied by the release of several digestive and absorptive hormones, some of which may contribute to any sensation of satiation, no obvious trigger for hunger was initially recognized. Then, in 2001, a gastric hormone ghrelin, a GH secretagogue, was found to be secreted synchronously with the initiation and termination of meals^10^. Neither this first report, nor the follow-up paper^11^ showing a 24% increase in meal-associated ghrelin concentrations after weight loss in subjects with obesity, and the hormone’s disappearance after gastric surgery, actually measured the appetite ratings. Yet, its general acceptance as a stimulus of hunger was a result of a report^12^ that infusions of ghrelin at 5 pM/kg/h resulting in supra-physiological plasma ghrelin concentrations, increased food intake and raised hunger scores at meal times^12^. Several questions related to acceptance of ghrelin as a cause of hunger remain. At the infusion rate 5 pM/kg/h and its supra-physiological concentrations, ghrelin elicited near maximal GH secretion^12^. Another GH secretagogue, GHRP-2, stimulated a 36% increase in food intake^13^, but this did not challenge the conclusion that ghrelin, rather than GH, stimulated hunger. Ghrelin may also affect hunger by participating in glucoregulation, energy homeostasis, cardioprotection, muscle atrophy, and bone metabolism^14^.

**Figure 1:**
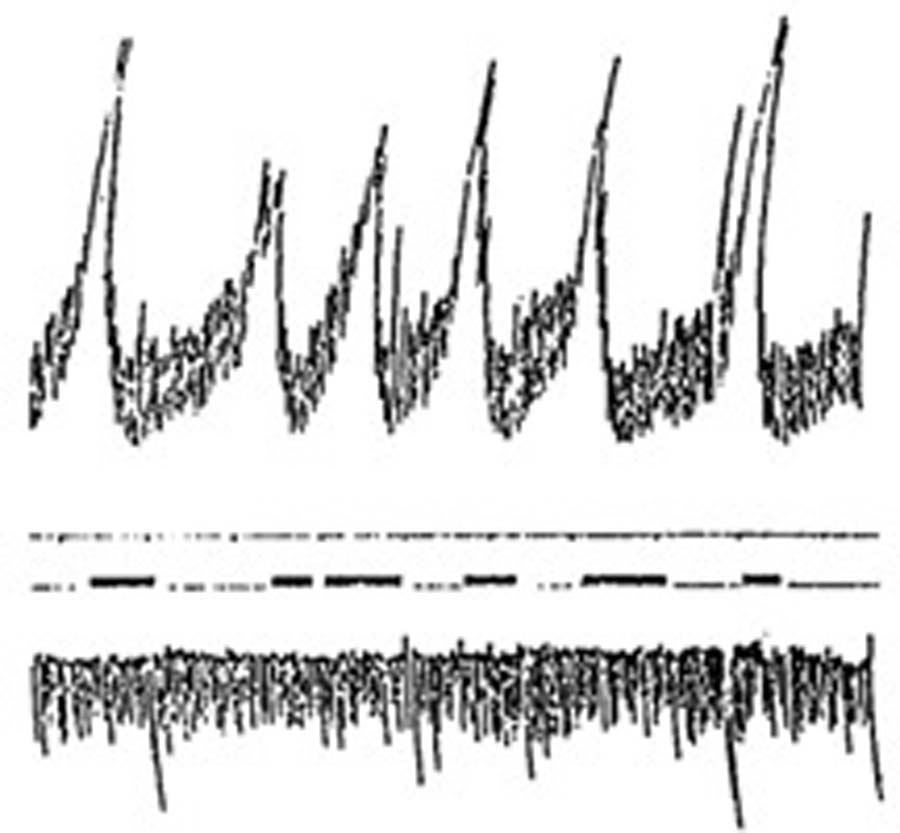
One half the original size. The top record represents intragastric pressure (the small oscillations due to respiration, the large to contractions of the stomach); the second record is time in minutes (ten minutes); the third record is W’s report of hunger pangs; the lowest record is respiration registered by means of a pneumograph about the abdomen.

Long forgotten was a classical 1912 study^15^ which demonstrated a close relationship between stomach contractions and reports of stomach hunger pangs. Dr. Washburn reported these in fasting state, as his stomach was equipped with a water-filled balloon and connected to a recorder which demonstrated to the attending Dr. Cannon a correspondence between the graphed stomach contractions and reported hunger pangs (Figure 1). Most well-nourished individuals will seldom reach a level of fasting sufficient to detect clear hunger pangs, but almost all of us are aware of an empty stomach after our meals have been digested.

### 2. Endogenous perception of satiation or fullness.

Gastric wall stretch and tension trigger afferent mechanoreceptor and vagal signals to the brain that produce a sensation of fullness in proportion to the volume of the ingested meal^16^. Sensation of fullness upon eating, and associated autonomic and hormonal messages to the brain, reduce and stop food intake as an immediate meal-associated negative feedback^17^. A number of studies attest to the dominant role of meal size and stomach stretch in the sensation of fullness. In an 11-week study^18^, healthy volunteers consumed identical meals differing only in energy density, containing either 30 to 35% fat, or 20 to 25% fat. Both groups consumed approximately the same daily volume of food (between 1400 and 1450 g) without adjusting the quantity eaten to the difference in dietary energy content. As a result, the body weights of the two groups diverged.

**Figure 2.**
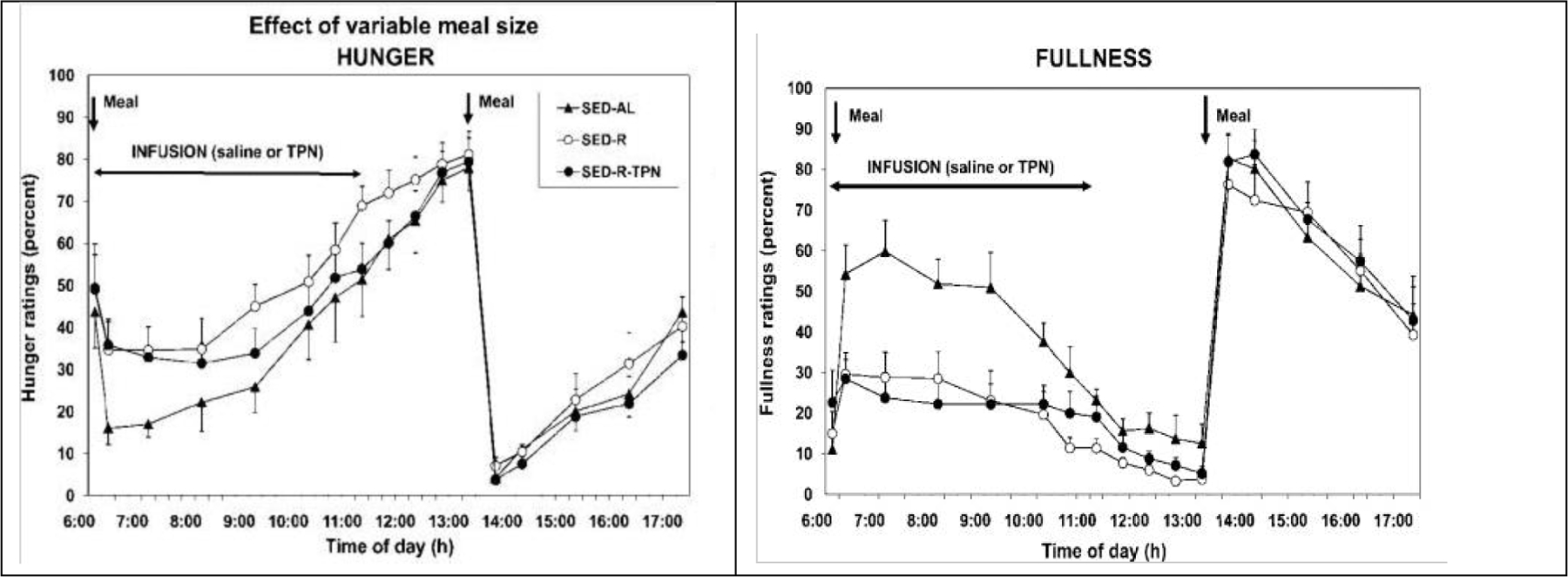
Hunger (left) and fullness (right) are detected only when orally eaten food is processed by the GI tract. Both sensations respond to the volume of the meal (open circles: SED-AL 500 Kcal, SED-R 100 kcal meal). Intravenously supplemented calories in the small meal (SED-R-TPN, solid circle, left panel) or calories expended by exercise (open rectangles) (TPN, solid rectangles, right panel) were not detected. Modified from Borer et al., 2009.

In studies employing different-size intragastric nutrient loads, satiation increased in proportion to the load volume and stomach stretch^19^. In the third line of research^20^, hunger was measured in response to meals of different size (100 vs 500 kcal) taken by mouth and to intravenous total parenteral (TPN) supplementation of small meals with missing calories. Hunger was also tested when the large meal was combined with exercise-induced energy expenditure that depleted almost all of ingested calories in the large meal, with or without intravenous supplementation. Only the size of meals, ingested by mouth and processed by the GI tract, influenced hunger and satiation (Figure 2). The 100 kcal meal elicited higher hunger and lower fullness than the 500 kcal meal. Supplementation of energy withheld from small meals or reduced by exercise, by intravenous nutrients did not influence either hunger or satiation. The dependence of hunger and sensation of fullness on the volume of food eaten by mouth and the GI transit of food is core evidence that oral and GI volumetric signals are the principal guides to human appetite.

### 3. Gut peptides primarily facilitate digestion and may incidentally control appetite.

Four gut peptides are considered to directly contribute either to hunger (ghrelin) or satiation (cholecystokinin, CCK, glucagon-like peptide 1, GLP-1, and peptide YY, PYY) in addition to controlling digestion by affecting GI physiology. Elicitation of hunger attributed to ghrelin was previously described. CCK arises from the duodenum, the proximal part of the small intestine, to stimulate gall bladder contractions and bile secretion for emulsification of dietary fat. It also affects pancreatic secretion and slows down gastric emptying. lntraperitoneal administration of CCK to sham-fed hungry rats^21^ stopped their feeding, and triggered a sensation of fullness in hungry humans after intragastric CCK infusion in combination with stomach-distending water balloons. Gall-bladder cramps were reported at higher concentrations^22^. Glucose-dependent insulinotropic peptide (GIP) and GLP-1 are incretins, hormones that enhance insulin secretion to glucose above what is seen with glucose ingestion alone^23,24^. GIP is secreted from upper, and GLP-1 from L cells in distal, small intestine. GIP also promotes energy storage in adipose tissue and osteoblast proliferation in bones. Like CCK, GLP-1 slows gastric emptying, but also suppresses glucose-dependent glucagon secretion. GLP-1 partners with peptide YY (PYY) to act as an ileal brake^24,25^. Both hormones are released from the enteroendocrine L cells in the distal gut responding to fat and to unabsorbed nutrients that reach ileum and colon. They change the peristaltic intestinal motility from propagative to segmenting and, by slowing GI nutrient transit, they allow proper nutrient absorption of large meals in the small intestine. Both hormones are also rapidly inactivated by the enzyme dipeptidyl peptidase IV (DPPIV). While the analogs of GLP-1 and PYY and the DPPIV inhibitors are utilized in medicine to suppress hunger and induce weight loss, the unresolved question is whether they cause satiation directly and independently of their effects on GI physiology.

**Figure 3.**
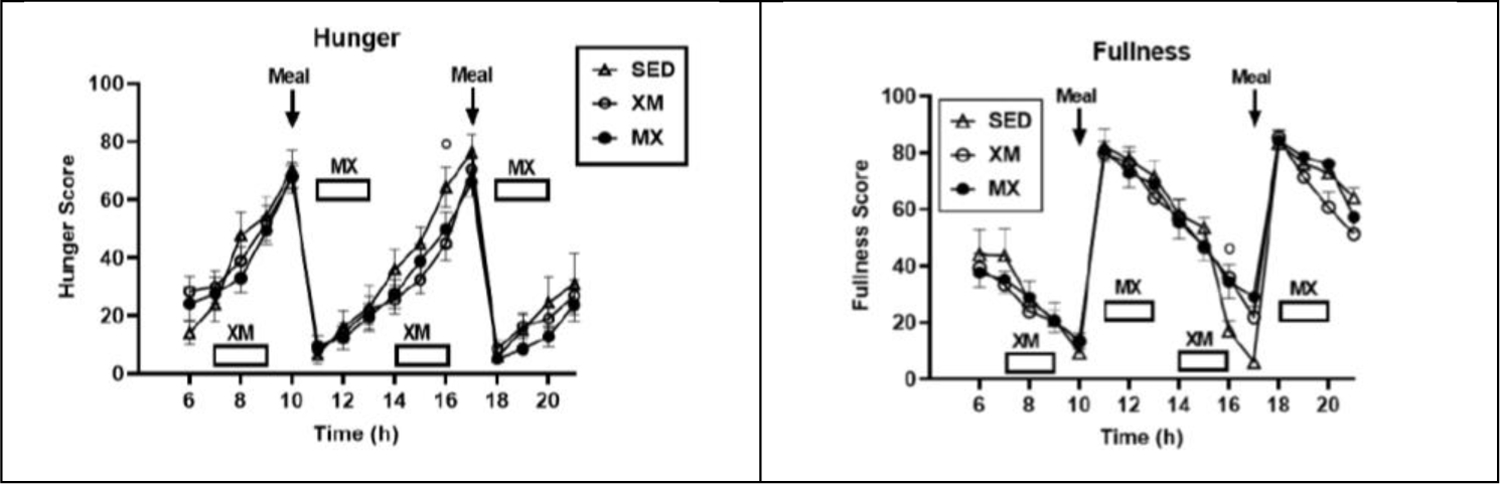
The course of hunger (left) and fullness (right) followed the timing of the meal transit through the GI tract and was largely unaffected by the timing of exercise with respect to meals. Modified after Borer et al., 2021.

To answer that question, the direct role of the five gut peptides in the control of human appetite was tested in a study^26^ where the secretion of ghrelin, GIP, GLP-1, PYY, and CCK were studied along with the meal-associated appetite ratings in response to energy-expending moderate-intensity exercise while fasted or during the meal-associated postprandial period. Subjects ate 1,600-kcals in two meals, at 10 and 17 h, and engaged in two hours of exercise starting either 1 h after (MX), or ending 1 h before eating (XM). We hypothesized that exercising fasted will elicit greater release of FFAs, ketone body beta-hydroxy butyrate (BHB), suppress GIP, GLP-1, PYY, CCK release and stimulate ghrelin and hunger compared to exercising after the meal. Exercising after the meal was hypothesized to elicit greater sensation of fullness and secretion of GIP, GLP-1, PYY, CCK, leptin and insulin, and lower secretion of ghrelin than exercising hungry. While differential timing of exercise with respect to meals produced expected changes in the circulating metabolic fuels, it did not substantially change the pattern of hunger and fullness as shown in Figure 3.

**Figure 4.**
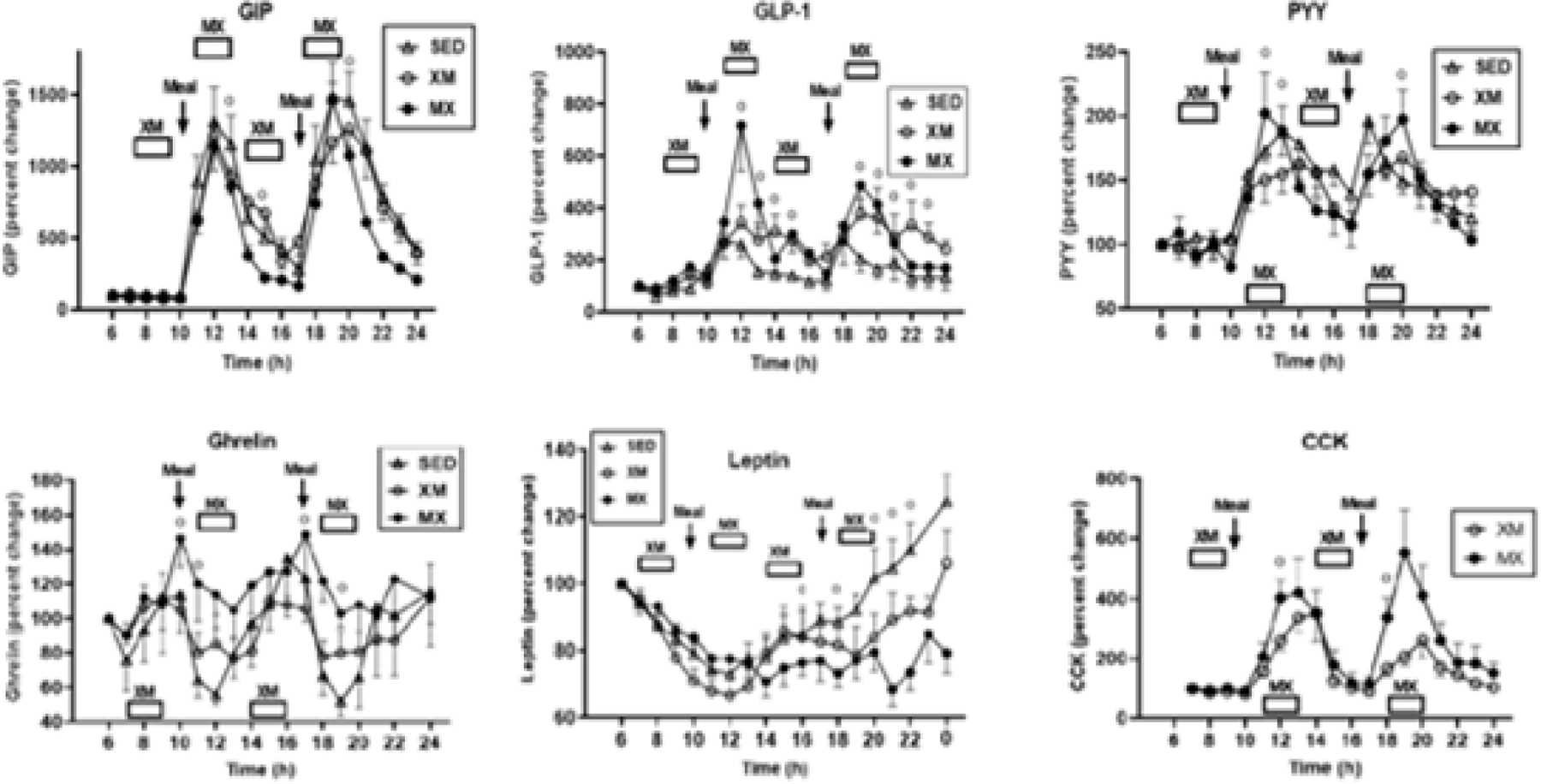
Concentrations of GIP, GLP-1, PYY (top panels) and ghrelin, and CCK (bottom panels) all increased during, and were timed by, digestion of the meals while being unaffected by timing of exercise with respect to meals. Only leptin concentrations responded to acute energy changes. MX= exercise after the meals, XM=Exercise before the meals, SED=sedentary controls. Modified from Borer et al., 2021

**Figure 5.**
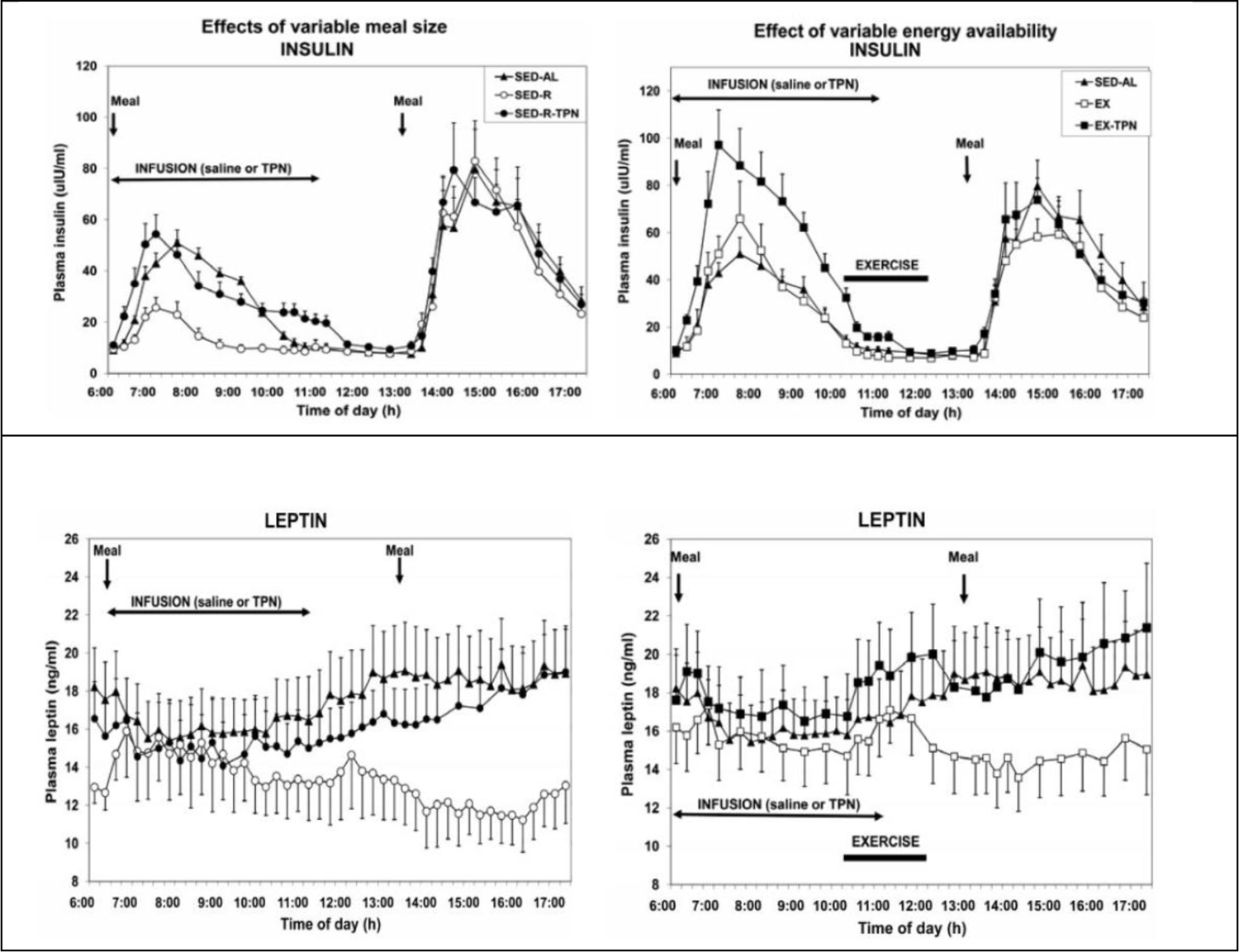
Left: Insulin (upper panels) and leptin respond (lower panels) to the energy content of the meals (500 kcal in SED-AL, solid triangles, 100 kcal small meals in SED-R, open circles) eaten by mouth and digested by GI tract, and 366 Kcals of intravenous TPN infusion (SED-R-TPN). Right: Both hormones responded to expenditure of 554 kcal exercising (EX, open rectangles) in addition to calories in the meals and TPN infusion. Modified from Borer et al., 2009.

The anticipated increases in hunger and plasma ghrelin when exercise preceded the meals (XM), and of GLP-1, PYY, and CCK when exercise was performed after eating (MX), did not take place. Ghrelin, GIP, GLP-1, PYY, and CCK concentrations followed the temporal pattern of meal digestion and absorption and were largely unaffected by the timing of meals and exercise. Only leptin concentration increased with ad libitum meals and was suppressed by energy expenditure of exercise. (Figure 4). In contrast to the five gut peptides, insulin and leptin track circulating calories^20^ (Figure 5), but also do not affect the appetite (Figure 2). Plasma insulin and leptin concentrations changed in proportion to the intravenous nutrient load, whether it was provided by different size of eaten meal, intravenously infused nutrients, or reduced by exercise energy expenditure (Figure 5)..

These studies^20,26^ support the primacy of the digestive role for GIP, GLP-1, PYY, ghrelin, and CCK in the course of the meal transit through the GI tract rather than a direct and independent effect on the appetite. Any contribution of gut peptides to fullness is incidental and most likely secondary to their effect on GI physiology.

### 4. Causes of human overeating.

**Figure 6.**
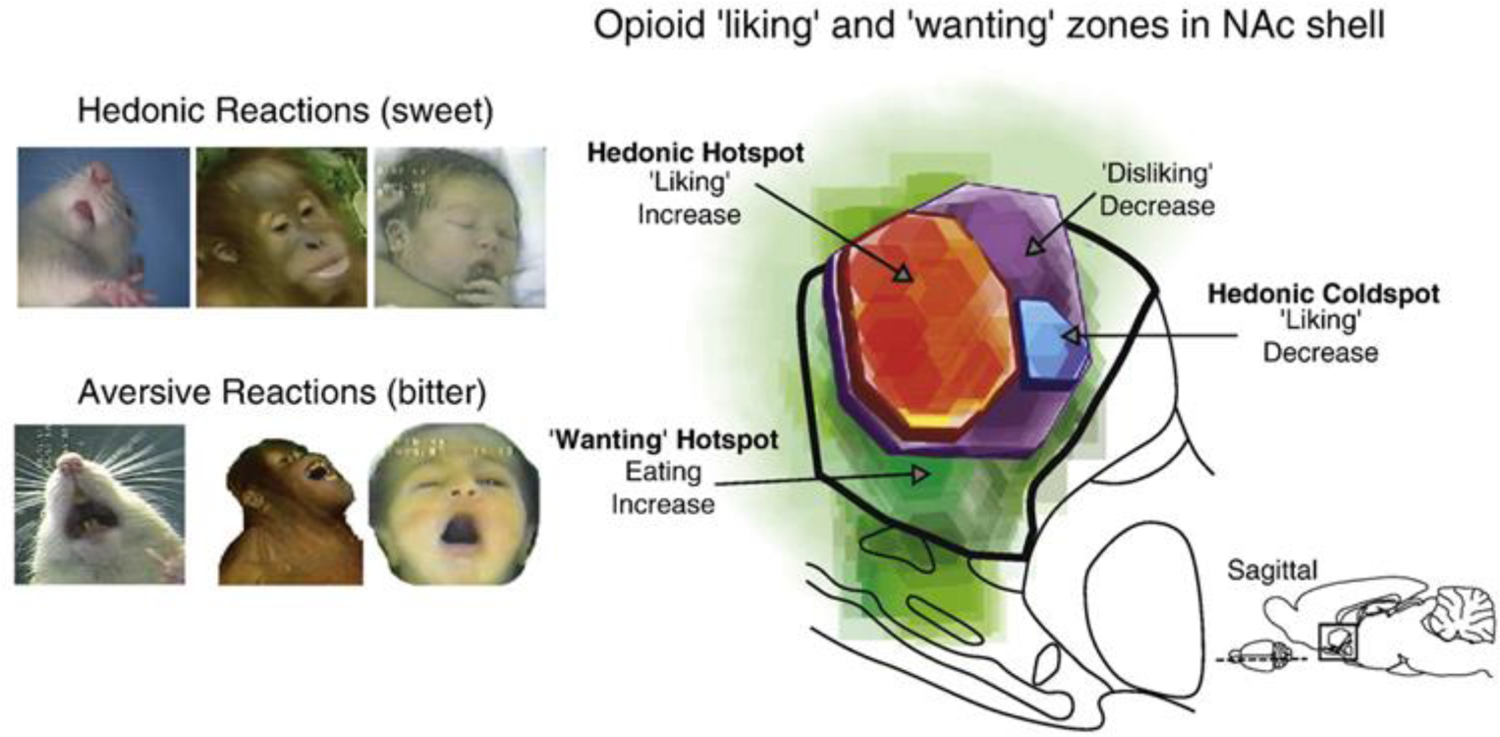
Hedonic “liking” reactions in human babies, primates, and rats (left) and a regions of rat nucleus accumbens that control hunger or “wanting” motivation and hedonic “liking” motivation. From Berridge 2009.

Two powerful endogenous causes that facilitate overeating are our inborn motivation to seek palatable taste and social facilitation, a behavior to eat more in the company of conspecifics whether they are humans^27^, dogs^28^, or newly hatched chicks^29^. Human hedonic motivation for sweet and savory foods is evident at birth as newborn babies smile to a sweet stimulus in their mouth, and cringe to acid and bitter tastes^30^ (Figure 5). The reason we seek any edible food when we are food deprived, and desire food that tastes and smells good at other times, is a brain mechanism that powers both kinds of motivation, “wanting” food when our stomachs are empty, and seeking food that we “like” even when we may be well fed^31^. Substantial orbitofrontal, insular cortical, and limbic circuits represent a substrate of the hunger motivation in response to negative energy balance. Interspersed within this circuitry, and centered in the mesolimbic nucleus accumbens, are neural substrates of hedonic motivation (Figure 6) which operate with dopamine and opioid neurotransmitters even under energy repletion.

We demonstrate our inborn liking of sweets by supporting development and marketing of sweet foods by food industry. Examples are doughnut shops, cake bakeries, and companies like Hershey Co, Mars Wrigley Confectionery, Nestle SA and many others. Beyond the motivation for palatability, food variety can enhance appeal and intake of food by as much as 29%^32^. Humans practice gastronomy and demonstrate the desire for palatability in what they cook and how they sequence palatable foods in their meals from salty and savory at the start of meals to palatable sweets at the end.

Social facilitation also leads to overeating in the company of others. The priming stimuli can include seeing other customers eating in restaurants^33^ or on television^34^, both of which can even influence the speed of eating^35^. It is likely that human opportunism, rather than an inborn trait, prompts people to eat more when offered a greater quantity of food at a lower price as in smorgasbord, fast-food and other restaurants^33,36, 37.^ Overeating also is encouraged when food is offered in oversize packages^38^.

Environmental and social incentives to overeat are present in extended periods of wakefulness in the current artificially illuminated world. Studies on the number of daily eating episodes (defined as ingestion of greater than 5 calories at a time), show that humans eat frequently and chaotically for up to 15 hours a day whether they are slightly overweight^39^, or overweight and obese^40^. Overabundance of fast-food and convenience food stores in most countries encourages easy access to palatable nutrients and influences preponderance of obesity^41,42^. In addition, competitive feeding contests such as a well-known “Hot-dog” or ‘Chicken-eating’’ contests encourage ingestion of supra-physiological amounts of food^43^.

A final unintended social incentive to overeat was promoted by Departments of Agriculture and Health and Human Services in 2010 with a recommendation, which has not been rescinded, that Americans consume between 45 and 65% of carbohydrates in their diets^44^. The recommendation was strongly influenced by the research of Ancel Keys in the 1950s with the objective to prevent development of atherosclerosis and CHD from high-fat diets^45^. The national recommendation probably facilitated weight gain by causing a 30.5% increase in daily carbohydrate consumption from 213 g per day in 1965 to 278 g per day or 51% of daily calories in 2011^46^. The currently high carbohydrate consumption falls within the 45 to 65% of daily calorie range as recommended since 2010^44^.

### 5. Causes of human inactivity.

**Figure 7.**
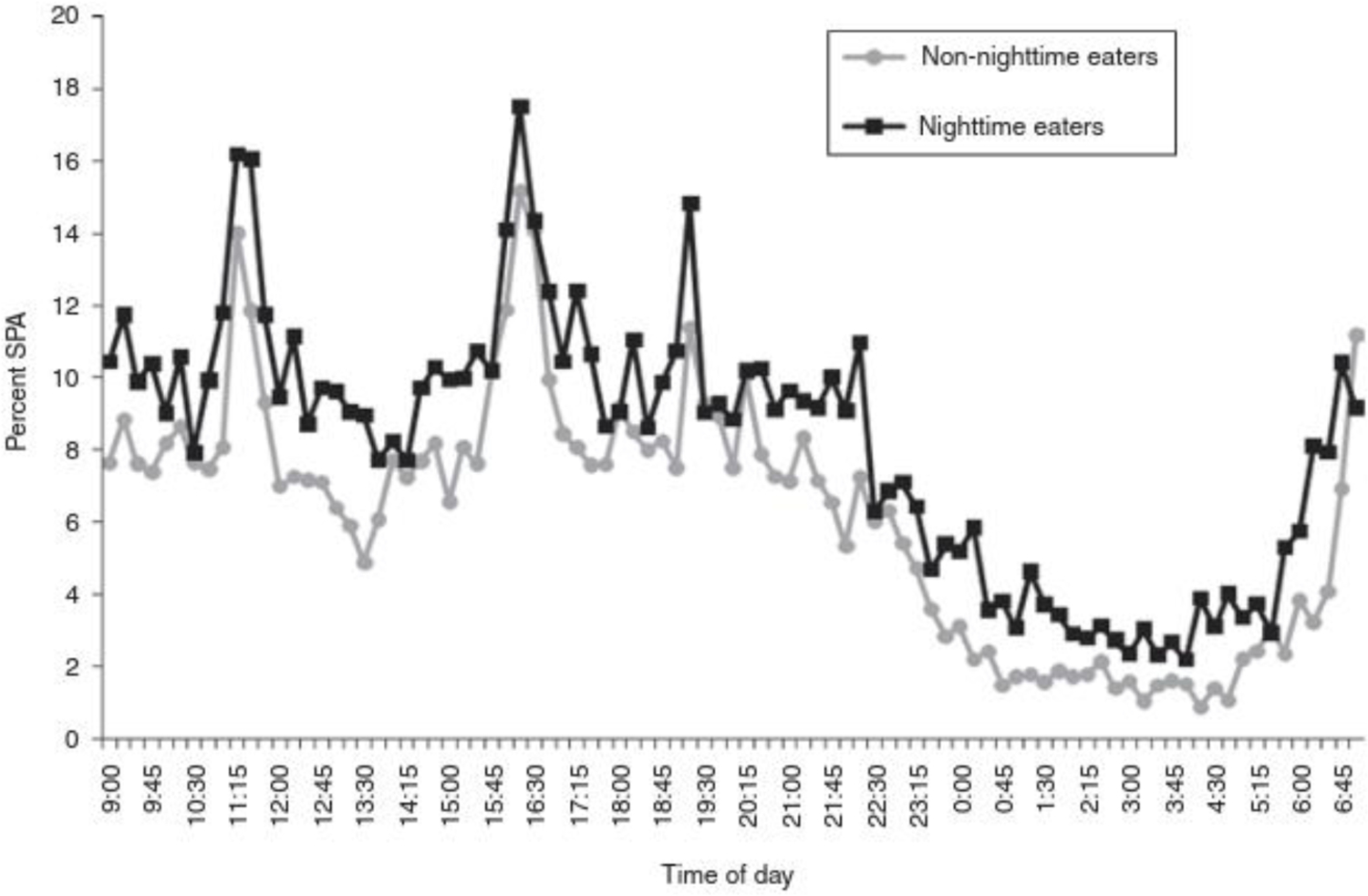
Percent of spontaneous physical activity in non-nighttime eater controls and nightime eaters during 24-h stay in the respiratory chamber. From Gluck et al., 2012.

Higher levels of physical activity are observed within half an hour prior to scheduled mid-day and evening meals provided at 11:30, 17:00 h, and a 20:00-h in healthy-eating subjects and in subjects with night eating disorder^47^(Figure 7). Short episodes of sleepiness are often reported 2 to 3 h after the large meals^48^ indicating a reciprocal relationships between short-term energy availability and spontaneous physical activity. Similarly, an acute increase in voluntary activity in rats anticipates restricted daily meals^49^ and was known since 1967.

**Figure 8.**
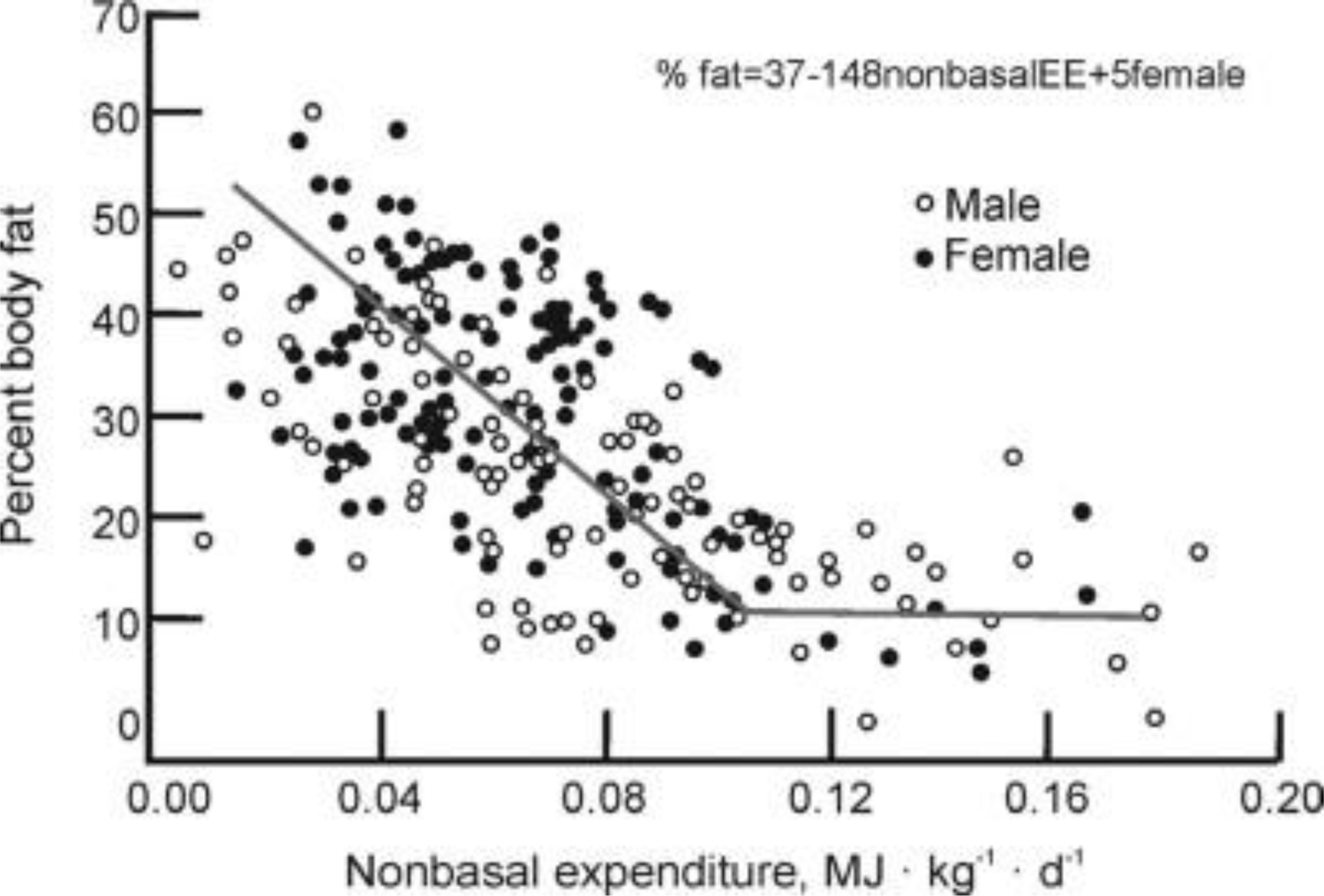
The inverse relationship between body fat and voluntary daily energy expenditure excluding the resting metabolic rate. Modified from Rising et al., 1994 and Schultz & Schoeller 1994.

That the relationship between momentary meal taking and spontaneous activity is not fortuitous but reflects an endogenous relationship between body energy status and the motivation to move is seen by examining the relationship between body fatness and the levels of spontaneous physical activity (Figure 8). As was the case for physical activity in relation to daily meals (Figure 7), the relationship between body fat and spontaneous physical activity is non-homeostatic, in that activity increases in normal-weight subjects and declines upon weight and fat gain^50,51^ (Figure 8).

Reasons for reduced energy expenditure in contemporary developed societies are also environmental and social, as they reflect human preference for technological aids and urban design that obviate the use of physical effort in daily living.

That the recent introduction of these conveniences has influenced physical work and body fatness is illustrated in a study of the lifestyle of an Old Order Amish community in Canada during 2004 planting season^52^. Unlike urban dwellers in developed societies, Amish rake leaves, milk cows by hand, hand-wash laundry and dishes, chop firewood for cooking and home heating, and grow, harvest, and can their food. Amish men averaged 10 h/week of vigorous work and 12 hours/week of walking generating over 18,000 steps /day and energy expenditure of 3100 kcal/day. Amish women cared for large families, did domestic and vigorous farm work for 3.4 h/week and 39 h/week of moderate physical work, expending 1,850 kcal/day. The incidence of obesity in their community was only 4% and of overweight 26%.

The introduction of motor transportation revealed a clear positive relationship between the frequency of its use and increases in overweight and obesity^53,54^. Urban planning also inadvertently encourages weight gain and reduced physical activity through single-use suburban environments that necessitate the use of automobiles^55^ as opposed to multiple-use urban environments that encourage walking and bicycling^56^.

### 6. Human genetic predisposition for body fat storage.

**Figure 9.**
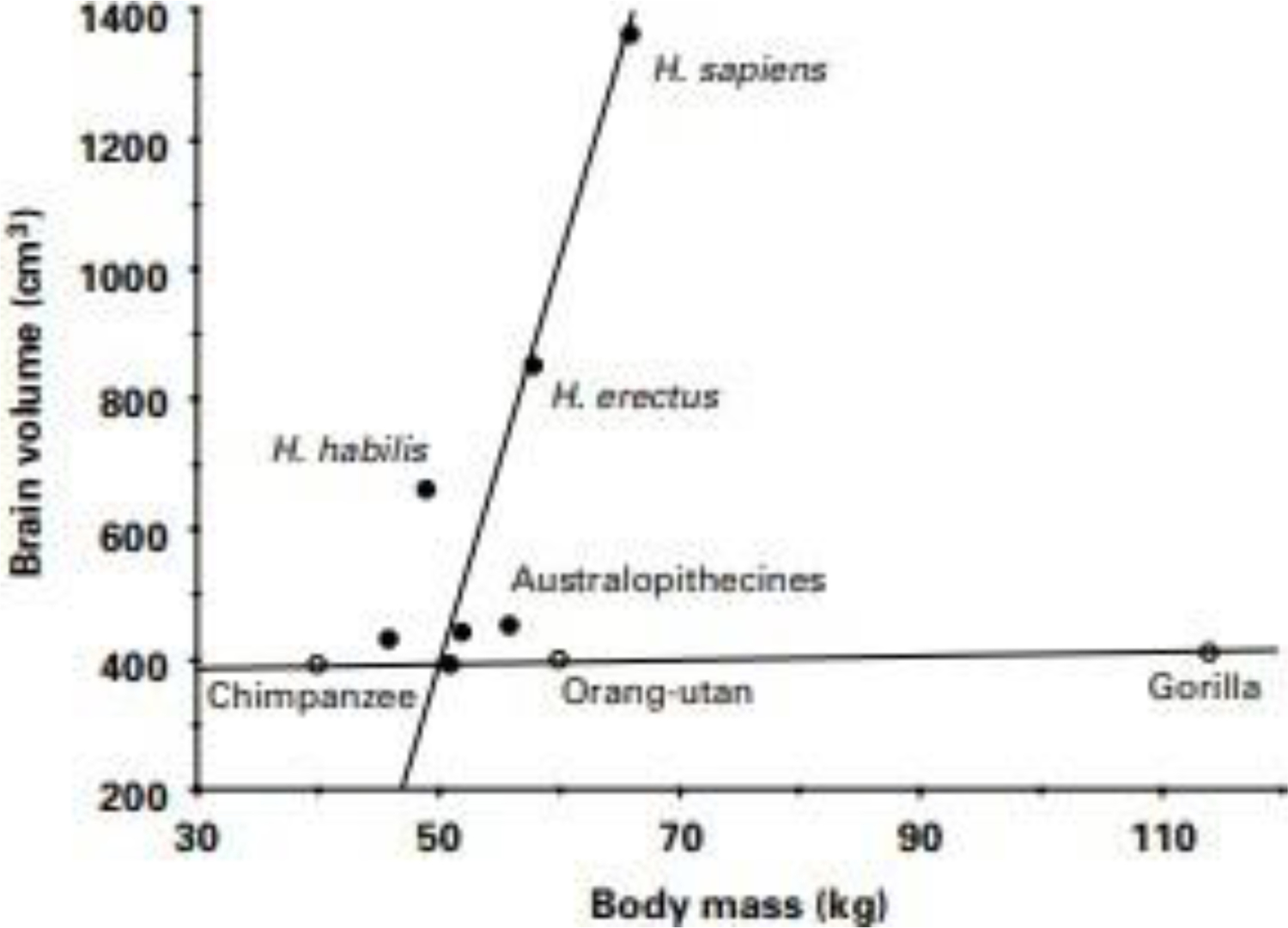
Increases in brain volume size in the hominid species. From Wells, 2006. Reproduced with permission.

Body fat in non-human primates averages between 5 and 10% of total body mass, in healthy men between 12 and 23%, and in women between 24 and 34%^57^.In individuals with obesity, body fat can reach upward of 50 to 60% (Figure 8), Increased capacity to store fat is hypothesized to have co-evolved with exponential development of brain size in the genus Homo over the past two million years^58^. The volume of human brain is more than three times the size of the brains in non-human primates (Figure 9).

It is speculated that the natural selection for increased fat storage and brain size co-evolved in response to seasonal fluctuations in energy resources in terrestrial savannah once humans developed bipedal locomotion. Larger brain consumes between 20 and 25% of resting metabolic rate and is benefited by larger fat stores. Evolution of increased adiposity in the human female^57^ supported higher energy cost of pregnancy, lactation, and feeding of multiple children.

Obesity is also a consequence of a genetic feed-forward, rather than feedback, operation of body mass control. Predisposition to seek palatable food whenever the stomach is empty and to eat chaotically over the extended wakeful period^39,40^ is balanced only by a temporary sensation of post-meal fullness, and not by any mechanism that would block overeating and increase energy-expending physical activity. This point is worth emphasizing as there is a scientific bias to look for a negative feedback mechanism in most life-supporting functions, such as regulation of stable blood glucose level. A homeostatic feedback hypothesis of regulation of body weight was proposed in 2000^59^, several years after the discovery of hormone leptin^60^. Subsequently, circulating leptin concentration was found to be proportional to the size of the subcutaneous fat depot^61^. The homeostatic negative feedback was proposed to operate between the adipose tissue volume and hypothalamic brain circuits controlling feeding and energy expenditure and mediated by circulating leptin concentrations. The feedback was hypothesized to work by suppressing hunger and increasing exercise energy expenditure in proportion to the size of subcutaneous fat depot and mediated by leptin concentrations. The reverse was supposed to take place when the volume of adipose tissue declined^59^. This hypothesis was not supported on several counts. First, in a large trial where obese individuals were injected with doses of leptin ranging from sub-threshold to supra-physiological levels, there was no effect on appetite or weight loss^62^. Second, leptin concentrations rise in both humans and animals in parallel with the rise in body fat mass, a clear demonstration that this hormone does not operate as a weight-normalizing negative-feedback signal. Third, daily fat mass changes are much too small to affect meal-to-meal hunger or levels of spontaneous physical activity.

**Figure 10:**
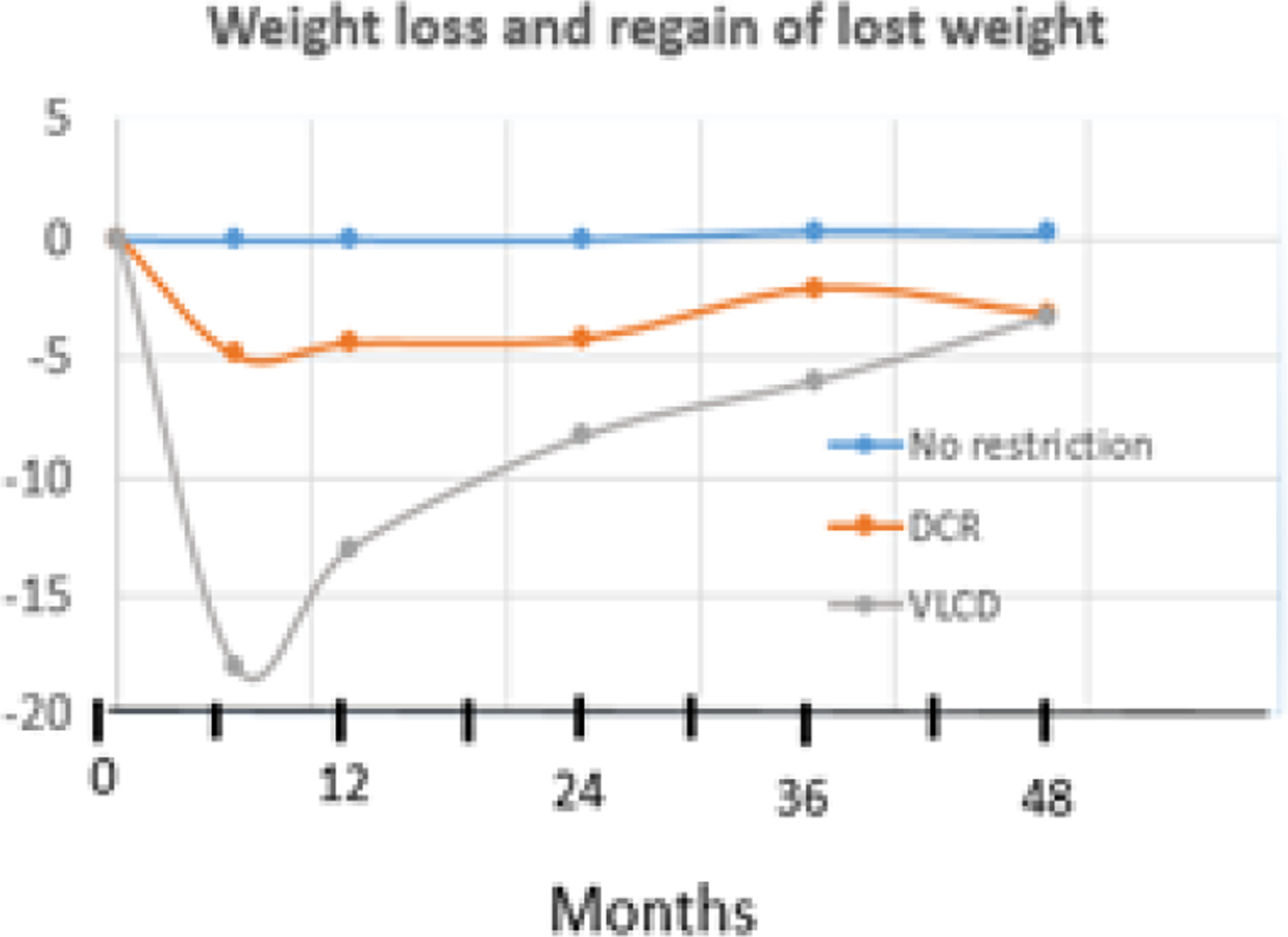
Percent of starting body weight lost during daily caloric restriction (DCR) and very-low calorie diet (VLCD) that is subsequently regained. Modified from Franz et al., 2007.

The third endogenous mechanism obstructing body fat loss includes increases in hunger and insulin sensitivity, along with reductions in energy expenditure through lower thyroid hormone titers, reduced sympathetic activity, and increased muscle work efficiency. The defense features appear to be mediated by declines in leptin concentrations during fasting as they are abolished by leptin injections to fasted and underweight individuals^63^. This defense mechanism is responsible for the relapse of obesity and regain of body weight after weight loss^7,8^ (figure 10):

In meal-to-meal eating, leptin released by the stomach^64^, rather than from the adipose tissue^65^, may modulate daytime energy intake and contribute to satiety by counter-regulating insulin secretion and action^66^. Individuals genetically unable to secrete leptin experience increased hunger and grow obese, but leptin administration corrects both abnormalities^67^. Yet, assertions that leptin “maintains the relative constancy of adipose tissue mass, thereby protecting individuals from the risks associated with being ... too obese”, persist^68^.

**Figure 11:**
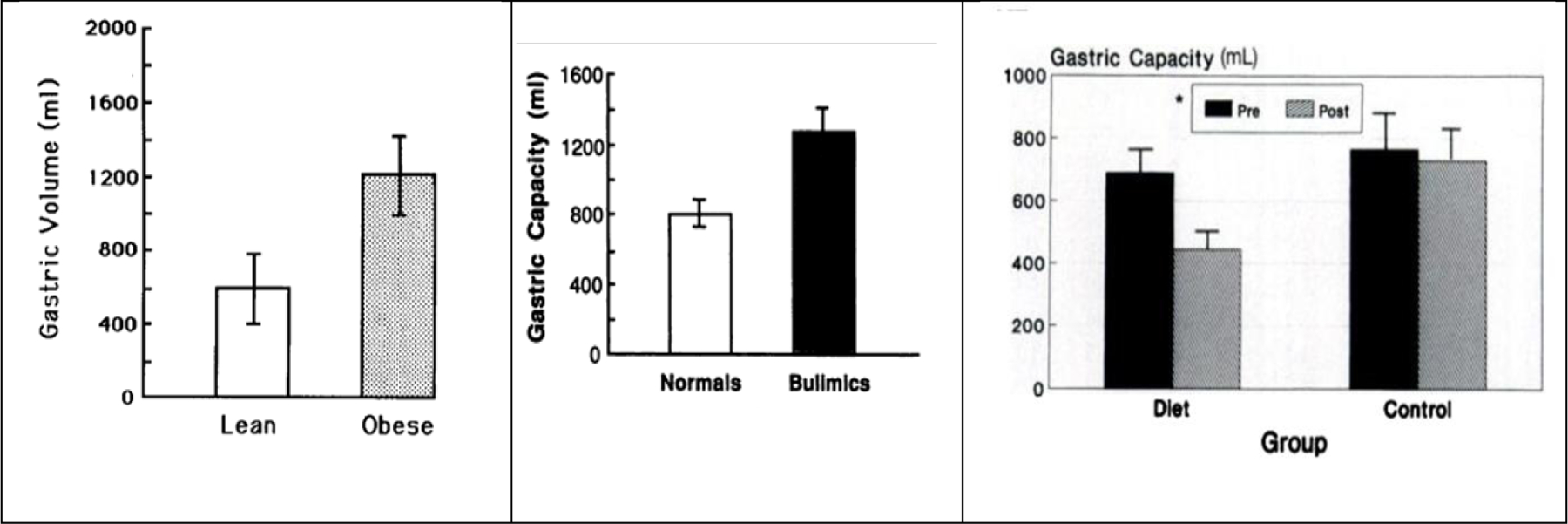
Changes in gastric volume as a function of obesity (left, Geliebter 1988), overeating in bulimia (center, Geliebter et al., 1992), and caloric restriction in subjects with obesity (right, Geliebter et al.,1996).

The final endogenous process that assists, rather than hinders, development of obesity is the capacity of stomach to hypertrophy in response to habitual overloading due to overeating^69^ (Figure 9). Measurements of stomach volume by water-filled balloons reveal that obese subjects^69^ and bulimic subjects^70^ have stomach volumes up to twice the size of normal-weight individuals (Figure 11) .

Meal intake correlated with, and stomach emptying was inversely related, to stomach size^70^, suggesting that enlarged gastric capacity may potentially provide a positive feedback for overeating. Individuals who habitually compete in annual Nathan’s Hot Dog Eating Contests can over 5 years increase their maximal consumption to more than 800 g/min in 10 minutes^43^ demonstrating the extent of large human GI plasticity. In contrast, a 4-week period of severe food restriction to 600 kcal/day produces a 27% reduction of gastric capacity^71^ (Figure 11, right). An even greater reduction in food consumption through a possible GI atrophy may have occurred in a case report where a 382-day absolute fast under medical supervision produced a massive weight loss followed by almost no weight gain over a subsequent 5 year period^72^

#### How best to manage body weight and energy balance?

Since the thesis of this review is that gastro-intestinal signals are the principal guides to human appetite and energy balance, how should individuals implement this guidance?. Here is a list of suggested options:

1. Resist environmental pressure to overeat whether caused by social facilitation during holidays, highly palatable foods, or commercial pressure to eat oversized portions in restaurants^73^. Follow “hara hachi bu” policy to eat only to 80% of stomach fullness as practiced by the long-lived Japanese on the island of Okinawa^74^;
2. Avoid habitual stomach overloading by overconsumption to prevent stomach hypertrophy^69,70^;
3. Be mindful of genetic obstacles to regulating body weight and body fat by substituting daily weighings of your body mass as a dependable low-tech feedback of management of energy balance^75^;
4. Incorporate deliberate physical activity (both aerobic locomotion and weight lifting) by embracing helpful technology of wearable activity monitors. They provide external motivation to exercise by stimulating our competitive impulses through step counts, energy expenditure feedback, and through intermittent encouragement to persist doing it;
5. Consider adding some features of intermittent fasting and meal eating to your regular routine. Time restricted eating^76^ counters human opportunistic tendency to snack whenever the opportunity presents itself^39,40^. Alternate-day fasting^77^ or a total fast on one, two, or three non-consecutive days during the week allow for mid-course corrections in the weight-control journey^78^.
6. Suggested recommendations work better in prevention than in correction of excess weight, but in either situation the outcome is likely to be better than doing nothing;
7. Finally, to oppose inborn features of human physiology and currently absent external support, national government and health organizations would need to provide counteracting external incentives for nationwide maintenance of healthy weight.

## CONCLUSIONS

Exponential rise of global obesity in the past three quarters of the century produced serious health and economic burdens. The prevailing approach to solving the obesity problem has been to consider obesity as an intractable disease amenable to pharmacological and surgical solutions, as most behavioral strategies have been unsatisfactory. The thesis of this review is (1) that these approaches are based on the faulty assumption that there is an innate energy- and weight-regulatory mechanism quantitatively controlling food intake and physical activity which malfunctions in some individuals predisposing them to inevitably become obese, but (2) that individuals have agency to prevent and attenuate obesity by understanding their inherited physiological limitations and predisposing social obstacles. They have the option to do so by avoiding social obstacles, deliberately attending to the signals from the oral and gastrointestinal tract to prevent overeating, and by using technological aids to frequently monitor their body weight and exploit the motivational features of activity monitors.
